# MUSCLE: a multiple sequence alignment method with reduced time and space complexity

**DOI:** 10.1186/1471-2105-5-113

**Published:** 2004-08-19

**Authors:** Robert C Edgar

**Affiliations:** 1 Department of Plant and Microbial Biology, 461 Koshland Hall, University of California, Berkeley, CA 94720-3102, USA

## Abstract

**Background:**

In a previous paper, we introduced MUSCLE, a new program for creating multiple alignments of protein sequences, giving a brief summary of the algorithm and showing MUSCLE to achieve the highest scores reported to date on four alignment accuracy benchmarks. Here we present a more complete discussion of the algorithm, describing several previously unpublished techniques that improve biological accuracy and / or computational complexity. We introduce a new option, MUSCLE-fast, designed for high-throughput applications. We also describe a new protocol for evaluating objective functions that align two profiles.

**Results:**

We compare the speed and accuracy of MUSCLE with CLUSTALW, Progressive POA and the MAFFT script FFTNS1, the fastest previously published program known to the author. Accuracy is measured using four benchmarks: BAliBASE, PREFAB, SABmark and SMART. We test three variants that offer highest accuracy (MUSCLE with default settings), highest speed (MUSCLE-fast), and a carefully chosen compromise between the two (MUSCLE-prog). We find MUSCLE-fast to be the fastest algorithm on all test sets, achieving average alignment accuracy similar to CLUSTALW in times that are typically two to three orders of magnitude less. MUSCLE-fast is able to align 1,000 sequences of average length 282 in 21 seconds on a current desktop computer.

**Conclusions:**

MUSCLE offers a range of options that provide improved speed and / or alignment accuracy compared with currently available programs. MUSCLE is freely available at .

## Background

Multiple alignments of protein sequences are important in many applications, including phylogenetic tree estimation, secondary structure prediction and critical residue identification. Many multiple sequence alignment (MSA) algorithms have been proposed; for a recent review, see [1]. Two attributes of MSA programs are of primary importance to the user: biological accuracy and computational complexity (i.e., time and memory requirements). Complexity is of increasing relevance due to the rapid growth of sequence databases, which now contain enough representatives of larger protein families to exceed the capacity of most current programs. Obtaining biologically accurate alignments is also a challenge, as the best methods sometimes fail to align readily apparent conserved motifs [2]. We recently introduced MUSCLE, a new MSA program that provides significant improvements in both accuracy and speed, giving only a summary of the algorithm [2]. Here, we describe the MUSCLE algorithm more fully and analyze its complexity. We introduce a new option designed for high-throughput applications, MUSCLE-fast. We also describe a new method for evaluating objective functions for profile-profile alignment, the iterated step in the MUSCLE algorithm.

### Current methods

**Figure 1:**
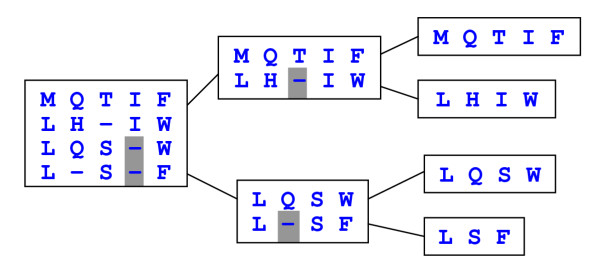
**Progressive alignment. **Sequences are assigned to the leaves of a binary tree. At each internal (i.e., non-leaf) node, the two child profiles are aligned using profile-profile alignment (see Figure 2). Indels introduced at each node are indicated by shaded background.

**Figure 2:**
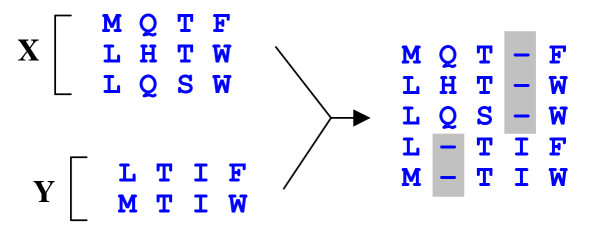
**Profile-profile alignment. **Two profiles (multiple sequence alignments) X and Y are aligned to each other such that columns from X and Y are preserved in the result. Columns of indels (gray background) are inserted as needed in order to align the columns to each other. The score for aligning a pair of columns is determined by the profile function, which should assign a high score to pairs of columns containing similar amino acids.

While multiple alignment and phylogenetic tree reconstruction have traditionally been considered separately, the most natural formulation of the computational problem is to define a model of sequence evolution that assigns probabilities to all possible elementary sequence edits and then to seek an optimal directed graph in which edges represents edits and terminal nodes are the observed sequences. This graph makes the history explicit (it can be interpreted as a phylogenetic tree) and implies an alignment. No tractable method for finding an optimal graph is known for biologically realistic models, and simplification is therefore required. A common heuristic is to seek a multiple alignment that maximizes the SP score (the summed alignment score of each sequence pair), which is NP complete [3]. It can be achieved by dynamic programming with time and space complexity O(*L*^*N*^) in the sequence length *L *and number of sequences *N *[4], and is practical only for very small *N*. Stochastic methods such as Gibbs sampling can be used to search for a maximum objective score [5], but have not been widely adopted. A more popular strategy is the progressive method [6,7] (Figure 1), which first estimates a phylogenetic tree. A profile (a multiple alignment treated as a sequence by regarding each column as a symbol) is then constructed for each node in the binary tree. If the node is a leaf, the profile is the corresponding sequence; otherwise its profile is produced by a pair-wise alignment of the profiles of its child nodes (Figure 2). Current progressive algorithms are typically practical for up to a few hundred sequences on desktop computers, the best-known of which is CLUSTALW [8]. A variant of the progressive approach is used by T-Coffee [9], which builds a library of both local and global alignments of every pair of sequences and uses a library-based score for aligning two profiles. On the BAliBASE benchmark [10,11], T-Coffee achieves the best results reported prior to MUSCLE, but has a high time and space complexity that limits the number of sequences it can align to typically around one hundred. In our experience, errors in progressive alignments can often be attributed to one of the following issues: sub-optimal branching order in the tree, scoring parameters that are not optimal for a particular set of sequences (especially gap penalties), and inappropriate boundary conditions (e.g., seeking a global alignment of proteins having different domain organizations). Misalignments are sometimes readily apparent, motivating further processing (*refinement*). One approach is to use a progressive alignment as the initial state of a stochastic search for a maximum objective score (*stochastic refinement*). Alternatively, pairs of profiles can be extracted from the progressive alignment and re-aligned, keeping the results only when an objective score is improved (*horizontal refinement*) [12].

## Implementation

The basic strategy used by MUSCLE is similar to that used by PRRP [13] and MAFFT [14]. A progressive alignment is built, to which horizontal refinement is then applied.

### Algorithm overview

MUSCLE has three stages. At the completion of each stage, a multiple alignment is available and the algorithm can be terminated.

### Stage 1: draft progressive

The first stage builds a progressive alignment.

#### Similarity measure

The similarity of each pair of sequences is computed, either using *k*-mer counting or by constructing a global alignment of the pair and determining the fractional identity.

#### Distance estimate

A triangular distance matrix is computed from the pair-wise similarities.

#### Tree construction

A tree is constructed from the distance matrix using UPGMA or neighbor-joining, and a root is identified.

#### Progressive alignment

A progressive alignment is built by following the branching order of the tree, yielding a multiple alignment of all input sequences at the root.

### Stage 2: improved progressive

The second stage attempts to improve the tree and builds a new progressive alignment according to this tree. This stage may be iterated.

#### Similarity measure

The similarity of each pair of sequences is computed using fractional identity computed from their mutual alignment in the current multiple alignment.

#### Tree construction

A tree is constructed by computing a Kimura distance matrix and applying a clustering method to this matrix.

#### Tree comparison

The previous and new trees are compared, identifying the set of internal nodes for which the branching order has changed. If Stage 2 has executed more than once, and the number of changed nodes has not decreased, the process of improving the tree is considered to have converged and iteration terminates.

#### Progressive alignment

A new progressive alignment is built. The existing alignment is retained of each subtree for which the branching order is unchanged; new alignments are created for the (possibly empty) set of changed nodes. When the alignment at the root is completed, the algorithm may terminate, return to step 2.1 or go to Stage 3.

### Stage 3: refinement

The third stage performs iterative refinement using a variant of tree-dependent restricted partitioning [12].

#### Choice of bipartition

An edge is deleted from the tree, dividing the sequences into two disjoint subsets (a bipartition). Edges are visiting in order of decreasing distance from the root.

#### Profile extraction

The profile (multiple alignment) of each subset is extracted from the current multiple alignment. Columns containing no residues (i.e., indels only) are discarded.

#### Re-alignment

The two profiles obtained in step 3.2 are re-aligned to each other using profile-profile alignment.

#### Accept/reject

The SP score of the multiple alignment implied by the new profile-profile alignment is computed. If the score increases, the new alignment is retained, otherwise it is discarded. If all edges have been visited without a change being retained, or if a user-defined maximum number of iterations has been reached, the algorithm is terminated, otherwise it returns to step 3.1. Visiting edges in order of decreasing distance from the root has the effect of first re-aligning individual sequences, then closely related groups

### Algorithm elements

In the following, we describe the elements of the MUSCLE algorithm. In several cases, alternative versions of these elements were implemented in order to investigate their relative performance and to offer different trade-offs between accuracy, speed and memory use. Most of these alternatives are made available to the user via command-line options. Four benchmark datasets have been used to evaluate options and parameters in MUSCLE: BAliBASE [10,11], SABmark [15], SMART [16-18] and our own benchmark, PREFAB [2].

### Objective score

**Figure 3:**
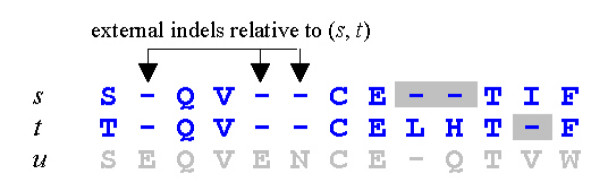
**Gap penalties in the SP score **This figure shows a multiple alignment of three sequences *s*, *t *and *u*. The SP score is the sum over all pairs of sequences of their pairwise alignment score. The contribution to the SP score from the pair (*s*, *t*) is computed by discarding columns in which both sequences have indels (arrows). Such indels are said to be external with respect to the pair. Gaps in the remaining columns (gray background) are assessed affine penalties *g *+ *λe *where *g *is the per-gap penalty, *λ *is the gap length, and *e *is the gap extension penalty.

In its refinement stage, MUSCLE seeks to maximize an objective score, i.e. a function that maps a multiple sequence alignment to a real number which is designed to give larger values to better alignments. MUSCLE uses the *sum-of-pairs *(SP) score, defined to be the sum over pairs of sequences of their alignment scores. The alignment score of a pair of sequences is computed as the sum of substitution matrix scores for each aligned pair of residues, plus gap penalties. Gaps require special consideration (Figure 3). We use the term *indel *for the symbol that indicates a gap in a column (typically a dash '-'), reserving the term *gap *for a maximal contiguous series of indels. The gap penalty contribution to SP for a pair of sequences is computed by discarding all columns in which both sequences have an indel, then applying an affine penalty *g *+ *λe *for each remaining gap where *g *is the per-gap penalty, *λ *is the gap length (number of indels in the gap), and *e *is the gap-length penalty (sometimes called the extension penalty).

### Progressive alignment

Progressive alignment requires a rooted binary tree in which each sequence is assigned to a leaf. The tree is created by clustering a triangular matrix containing a distance measure for each pair of sequences. The branching order of the tree is followed in postfix order (i.e., children are visited before their parent). At each internal node, profile-profile alignment is used to align the existing alignments of the two child subtrees, and the new alignment is assigned to that node. A multiple alignment of all input sequences is produced at the root node (Figure 1).

### Similarity measures

We use the term *similarity *for a measure on a pair of sequences that indicates their degree of evolutionary divergence (the sequences are assumed to be related). MUSCLE uses two types of similarity measure: the fractional identity *D *computed from a global alignment of the two sequences, and measures obtained by *k*-mer counting. A *k*-mer is a contiguous subsequence of length *k*, also known as a word or *k*-tuple. Related sequences tend to have more *k*-mers in common than expected by chance, provided that *k *is not too large and the divergence is not too great. Many sequence comparison methods based on *k*-mer counting have been proposed in the literature; for a review, see [19]. The primary motivation for these measures is improved speed as no alignment is required. MAFFT uses *k*-mer counting in a compressed alphabet (i.e., an alphabet in which symbols denote classes that may contain two or more residue types) to compute its initial distance measure. The alphabet used in MAFFT is taken from [20], and is one of the options implemented in MUSCLE. Trivially, identity is higher or equal in a compressed alphabet; it cannot be reduced. If the alphabet is chosen such that there are high probabilities of intra-class substitution and low probabilities of inter-class substitution, then we might expect that detectable identity (and hence the number of conserved *k*-mers) could be usefully extended to greater evolutionary distances while limiting the increase in matches due to chance. We have previously shown [21] that *k*-mer similarities correlate well with fractional identity, although we failed to find evidence that compressed alphabets have superior performance to the standard alphabet at lower identities. We define the following similarity measure between sequences X and Y:

*F *= Σ_*τ *_min [*n*_X_(*τ*), *n*_Y_(*τ*) ] / [min (*L*_X_, *L*_Y_) - *k *+ 1 ].     (1)

Here *τ *is a *k*-mer, *L*_X_, *L*_Y _are the sequence lengths, and *n*_X_(*τ*) and *n*_Y_(*τ*) are the number of times *τ *occurs in X and Y respectively. This definition can be motivated by considering an alignment of X to Y and defining the similarity to be the fraction of *k*-mers that are conserved between the two sequences. The denominator of *F *is the maximum number of *k*-mers that could be aligned. Note that if a given *k*-mer occurs more often in one sequence than the other, the excess cannot be conserved, hence the minimum in the numerator. The definition of *F *is an approximation in which it is assumed that (after correcting for excesses) common *k*-mers are always alignable to each other. MUSCLE also implements a binary approximation *F*^Binary^, so-called because it reduces the *k*-mer count to a present / absent bit:

*F*^Binary ^= Σ_*τ *_*δ*_XY_(*τ*) / [min (*L*_X_, *L*_Y_) - *k *+ 1 ].     (2)

Here, *δ*_XY_(*τ*) is 1 if *τ *is present in both sequences, 0 otherwise. As multiple instances of a given *k*-mer in one sequence are relatively rare, this is often a good approximation to *F*. The binary approximation enables a significant speed improvement as the size of the count vector for a given sequence can be reduced by an order of magnitude. This allows the count vector for every sequence to be retained in memory, and pairs of vectors to be compared efficiently using bit-wise instructions. When using an integer count, there may be insufficient memory to store all count vectors, making it necessary to re-compute counts several times for a given sequence.

### Distance measures

Given a similarity value, we wish to estimate an additive distance measure. An additive measure distance measure d(A, B) between two sequences A and B satisfies d(A, B) = d(A, C) + d(C, B) for any third sequence C, assuming that A, B and C are all related. Ideal but generally unknowable is the *mutation distance*, i.e. the number of mutations that occurred on the historical path between the sequences. The historical path through the phylogenetic tree extends from one sequence to the other via their most recent common ancestor. The mutation distance is trivially additive. The fractional identity *D *is often used as a similarity measure; for closely related sequences 1 - *D *is a good approximation to a mutation distance (it is exact assuming substitution at a single site to be the only allowed type of mutation and that no position mutates more than once). As sequences diverge, there is an increasing probability of multiple mutations at a single site. To correct for this, we use the following distance estimate [22]:

*d*_Kimura _= -log_e _(1 - *D *- *D*^2^/5)     (3)

For *D *≤ 0.25 we use a lookup table taken from the CLUSTALW source code. For *k*-mer measures, we use:

*d*_kmer _= 1 - *F*.     (4)

### Tree construction

Given a distance matrix, a binary tree is constructed by clustering. Two methods are implemented: neighbor-joining [23], and UPGMA [24]. MUSCLE implements three variants of UPGMA that differ in their assignment of distances to a new cluster. Consider two clusters (subtrees) *L *and *R *to be merged into a new cluster *P*, which becomes the parent of *L *and *R *in the binary tree. Average linkage assigns this distance to a third cluster *C*:

*d*^Avg^_*PC *_= (*d*_*LC *_+ *d*_*RC*_)/2.     (5)

We can take the minimum rather than the average:

*d*^Min^_*PC *_= min [*d*_*LC*_, *d*_*RC*_].     (6)

Following MAFFT, we also implemented a weighted mixture of minimum and average linkage:

*d*^Mix^_*PC *_= (1 - *s*) *d*^Min^_*PC *_+ *s d*^Avg^_*PC*_,     (7)

where *s *is a parameter set to 0.1 by default. Clustering produces a pseudo-root (the last node created). We implemented two other methods for determining a root: minimizing the average branch weight [25], as used by CLUSTALW, and locating the root at the center of the longest span.

### Sequence weighting

Conventional wisdom holds that sequences should be weighted to correct for the effects of biased sampling from a family of related proteins; however, there is no consensus on how such weights should be computed. MUSCLE implements the following sequence weighting schemes: none (all sequences have equal weight), Henikoff [26], PSI-BLAST [27] (a variant of Henikoff), CLUSTALW's, GSC [28], and the three-way method [29]. We found the use of weighting to give a small improvement in benchmark accuracy results, e.g. approximately 1% on BAliBASE, but saw little difference between the alternative schemes. The CLUSTALW method enables a significant reduction in complexity (described later), and is therefore the default choice.

### Profile functions

In order to apply pair-wise alignment methods to profiles, a scoring function must be defined for a pair of profile positions, i.e. a pair of multiple alignment columns. This function is the profile analog of a substitution matrix; see for example [30]. We use the following notation. Let *i *and *j *be amino acid types, *p*_*i *_the background probability of *i*, *p*_*ij *_the joint probability of *i *and *j *being aligned to each other, *S*_*ij *_the substitution matrix score, *f *^*x*^_*i *_the observed frequency of *i *in column *x *of the first profile, *f *^*x*^_G _the observed frequency of gaps in that column, and *α*^*x*^_*i *_the estimated probability of observing *i *in position *x *in the family. (Similarly for position *y *in the second profile). Estimated probabilities *α *are derived from the observed frequencies *f*, typically by adding heuristic pseudo-counts or by using Bayesian methods such as Dirichlet mixture priors [31]. A commonly used profile function is the sequence-weighted sum of substitution matrix scores for each pair of letters, selecting one from each column (PSP, for profile SP):

PSP^*xy *^= Σ_*i *_Σ_*j *_*f *^*x*^_*i *_*f *^*y*^_*j *_*S*_*ij*_.     (8)

Note that *S*_*ij *_= log (*p*_*ij *_/ *p*_*i*_*p*_*j*_) [32], so

PSP^*xy *^= Σ_*i *_Σ_*j *_*f *^*x*^_*i *_*f *^*y*^_*j *_log (*p*_*ij *_/ *p*_*i *_*p*_*j*_).     (9)

PSP is the function used by CLUSTALW and MAFFT. It is a natural choice when attempting to maximize the SP objective score: if gap penalties are neglected, maximizing PSP maximizes SP under the constraint that columns in each profile are preserved. (This follows from the observation that the contribution to SP from a pair of sequences in the same profile is the same for all alignments allowed under the constraint). MUSCLE implements PSP functions based on the 200 PAM matrix of [33] and the 240 PAM VTML matrix [34]. In addition to PSP, MUSCLE implements a function we call the *log-expectation *(LE) score. LE is a modified version of the log-average (LA) profile function that was proposed on theoretical grounds [35]:

LA^*xy *^= log Σ_*i *_Σ_*j *_*α*^*x*^_*i *_*α*^*y*^_*j *_*p*_*ij *_/ *p*_*i *_*p*_*j*_.     (10)

LE is defined as follows:

LE^*xy *^= (1 - *f *^*x*^_*G*_) (1 - *f *^*y*^_G_) log Σ_*i *_Σ _*j *_*f *^*x*^_*i *_*f *^*y*^_*j *_*p*_*ij *_/ *p*_*i*_*p*_*j*_.     (11)

The MUSCLE LE function uses probabilities computed from VTML 240. Note that estimated probabilities *α *in LA are replaced by observed frequencies *f *in LE. The factor (1 - *f*_G_) is the *occupancy *of a column. Frequencies *f*_*i *_must be normalized to sum to one if indels are present (otherwise the logarithm becomes increasingly negative with increasing numbers of gaps even when aligning conserved or similar residues). The occupancy factors are introduced to encourage more highly occupied columns (i.e., those with fewer gaps) to align, and are found to significantly improve accuracy. We avoid these complications in the PSP score by computing frequencies in a 21-letter alphabet (amino acids + indel), and by defining the substitution score of an amino acid to an indel to be zero. This has the desired effect of down-weighting column pairs with low occupancies, and can also be motivated by consideration of the SP function. If gap penalties are ignored, then this definition of PSP preserves the optimization of SP under the fixed-column constraint by correctly accounting for the reduced number of residue pairs in columns containing gaps.

### Gap penalties

**Figure 4:**
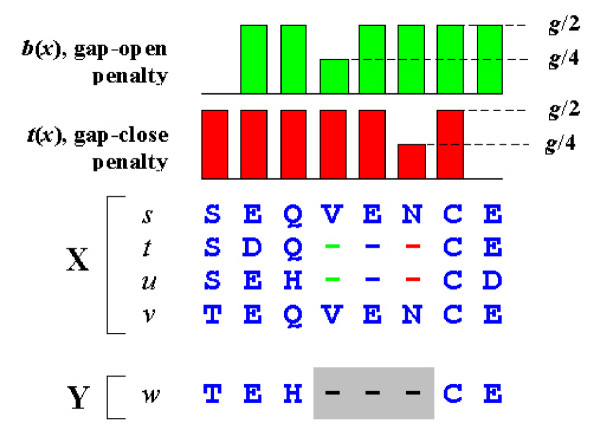
**Position-specific gap penalties. **An alignment of two profiles X and Y. Gaps in sequences *t *and *u *are embedded in X. Y contains a single sequence *w*. The gap in *w *(gray background) is inserted to align the profiles and is not part of Y. Consider the SP score for this alignment. We need not consider pairs of sequences in X as their scores are unchanged under all possible alignments of X to Y, leaving the inter-profile pairs (*s*, *w*), (*t*, *w*), (*u*, *w*) and (*v*, *w*). Note that there is no gap penalty for the pairs (*u*, *w*) and (*v*, *w*) as these pairs do not have gaps relative to each other. The remaining pairs (*t*, *w*) and (*u*, *w*) are assessed a penalty *g *+ 3*e *for the gap in Y. The total over all pairs of open or close penalties due to a gap in Y is thus reduced in proportion to the fraction of sequences in X having a gap with the same open or close position. We incorporate this into the PSP score by using position-specific gap penalties *b*(*x*) and *t*(*x*). For example, *b*(*x*) in column 4 of X is half the default value because half of the sequences in X open a gap in that column. Note that there is no open penalty at the N-terminal and no close penalty at the C-terminal. This causes terminal gaps to receive half the penalty of internal gaps.

We call the first indel in a gap its *gap-open*; the last its *gap-close*. Consider an alignment of two profiles X and Y, and a gap of length *λ *in X in which the gap-open is aligned to position *y*_*o *_in Y and the gap-close to *y*_*c*_. The penalty for this gap is *b*(*y*_*o*_) + *t*(*y*_*c*_) + *λe*, where *b *and *t *are costs for opening and closing a gap that vary according to the position in Y, and *e *is a length cost (sometimes called a gap extension penalty) that does not vary by position. A fixed length cost allows a minor optimization of the scoring scheme [14]. Consider a global alignment of sequences X and Y having lengths *L*_X _and *L*_Y_. If a constant *δ *(the *center*) is added to each substitution matrix score and *δ*/2 is added for each gapped position, this adds the constant value *δ*(*L*_X _+ *L*_Y_)/2 to the score of any possible alignment, and the set of optimal alignments is therefore unchanged. Given a scoring scheme with substitution matrix *S*_*ij *_and extension penalty *e*, we can thus choose *δ*/2 = *e *and instead use *S'*_*ij *_= *S*_*ij *_+ 2*e *and *e' *= 0 to obtain the same alignment. The constant 2*e *can be added to the substitution matrix at compile time, and no explicit extension penalty is then needed in the recursion relations. MUSCLE uses this optimization for the PSP function, but not for LE (where the center must be added at execution time after taking the logarithm). Let *f *^*y*^_*o *_be the number of gap-opens in column *y *in Y and *f *^*y*^_*c *_be the number of gap-closes in column *y*. MUSCLE computes *b *and *t *as follows (Figure 4):

*b*(*y*) = *g*/2 (1 - *f *^*y*^_*o*_) (1 + *h*_*w*_(*y*)*H*),     (12)

*t*(*y*) = *g*/2 (1 - *f *^*y*^_*c*_) (1 + *h*_*w*_(*y*)*H*).     (13)

Here, *g *is a parameter that can be considered a default per-gap penalty, *h*_*w*_(*y*) is 1 if *y *falls within a window of *w *consecutive hydrophobic residues or zero otherwise, and *H *is a tunable parameter. By default, *w *= 5, *H *= 1.2. The factor *g*/2 (1 - *f *^*y*^_*o*_) is motivated by considering the SP score of the alignment. The gap penalty contribution to SP for a pair of sequences (*A *∈ Y, *B *∈ X) is computed by discarding all columns in which both sequences have an indel, then applying an affine penalty *g *+ *λe *for each remaining gap. It is convenient here to consider that half of the per-gap penalty *g *is applied to the open position and half to the close position. Suppose a gap *G *is inserted into X such that the gap-open is aligned to position *y *in Y. If a sequence *s *∈ Y has a gap-open at *y*, then the SP score includes no open penalty for *G *induced by any pair (*s*, *t*) : *t *∈ X. The multiplier (1 - *f *^*y*^_*o*_) therefore corrects the gap-open contribution to the SP score due to pre-existing gaps in Y. (It should be noted that even with this correction, there are other issues related to gaps and PSP still does not exactly optimize SP under the fixed-column constraint). The increased penalty in hydrophobic windows is designed to discourage gaps in buried core regions where insertions and deletions are less frequent. Note that MUSCLE treats open and close positions symmetrically, in contrast to CLUSTALW, which treats the open position specially and may therefore tend to produce, in word processing terms, left-aligned gaps with a ragged right margin.

### Terminal gaps

A *terminal gap *is one that opens at the N-terminal position of the sequence to which it is aligned or closes at the C-terminal; as opposed to an *internal gap*. It has been suggested [9,36] that global methods have intrinsic difficulties with long deletions or insertions. We believe that these difficulties are often due to the choice of penalties for terminal gaps. CLUSTALW, which charges no penalty for terminal gaps, tends to fail to open a needed internal gap and thus fail to align terminal motifs; MAFFT, which charges the same penalty for terminal and internal gaps, sometimes aligns small numbers of residues to a terminal by inserting an unnatural internal gap. By default, MUSCLE penalizes terminal gaps with half the penalty of an internal gap. This is done by setting *b*(1), the open penalty at the C-terminal, and *t*(*L*), the close penalty at the N-terminal, to zero (Figure 4). The option of always applying full penalties, as in MAFFT, is also provided. We found that the compromise of a half penalty for terminal gaps gave good results for a wide range of input data, but that further improvements could sometimes by achieved by the following technique. If the length ratio of the two profiles to be aligned exceeds a threshold (by default, 20%), then MUSCLE constructs four different alignments in which gaps at both, one or neither terminals are fully penalized. A *conservation score *is defined by subtracting all gap penalties (both internal and terminal) from the alignment score, leaving a sum over profile functions only. The alignment with the highest conservation score is used.

### Tree comparison

**Figure 5:**
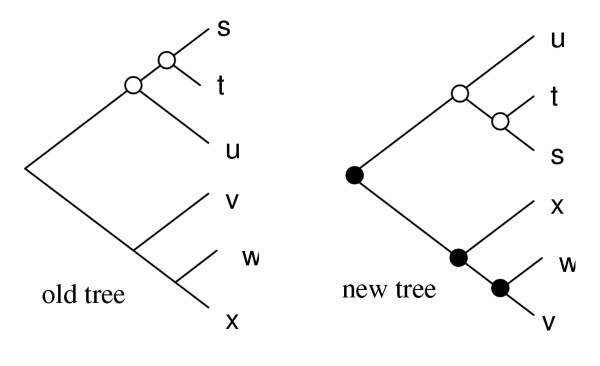
**Tree comparison. **Two trees are compared in order to identify those nodes that have the same branching orders within subtree rotation (white). If a progressive alignment has been created using to the old tree, then alignments at these nodes can be retained as the same result would be produced at those nodes by the new tree. New alignments are needed at the changed (black) nodes only.

In progressive alignment, two subtrees will produce identical alignments if they have the same set of sequences at their leaves and the same branching orders (topologies). We exploit this observation to optimize the progressive alignment in Stage 2 of MUSCLE, which begins by constructing a new tree. Unchanged subtrees are identified, and their alignments are retained (Figure 5). A progressive alignment of the changed subtrees is constructed, producing the same alignment at the root that would be obtained starting from the leaves. Tree comparison is performed by the following algorithm. Consider two trees *A *and *B *with identical sets of *N *leaves. Leaves are identified by consecutive integers (*ids*) 1 through *N*. Call a pair of nodes, one from each tree, equivalent if they are the same leaf or they are internal nodes and their children are equivalent. The left/right position of a child is not considered; in other words, subtree rotations are allowed (because they do not change the results of a progressive alignment). Traverse *A *in prefix order (children before their parent), assigning internal nodes ids *N *+ 1 through 2*N *in the order visited. When visiting an internal node *P*^*A*^, take the ids of its two child nodes *L*^*A *^and *R*^*A *^and use them as indexes into a lookup table pointing to nodes in *B*. If (a) *L*^*A *^is equivalent to a node *L*^*B *^in *B *and *R*^*A *^is equivalent to a node *R*^*B*^, and (b) *L*^*B *^and *R*^*B *^have the same parent *P*^*B*^, then assign *P*^*B *^the same id as *P*^*A*^, to which it is equivalent. When the traversal is complete, a node *b *in *B *is equivalent to some node in *A *if and only if *b *has an id. This procedure is O(*N*) time and space.

### Defaults, optimizations and complexity analysis

We now discuss the default choices of algorithm elements in the MUSCLE program and analyze their complexity.

### Complexity of CLUSTALW

It is instructive to consider the complexity of CLUSTALW. This is of intrinsic interest as CLUSTALW is currently the most widely used MSA program and, to the best of our knowledge, its complexity has not previously been stated correctly in the literature. It is also useful as a baseline for motivating some of the optimizations used in MUSCLE. The CLUSTALW algorithm can be described by the same steps as Stage 1 above. The similarity measure is the fractional identity computed from a global alignment, clustering is done by neighbor-joining. Global alignment of a pair of sequences or profiles is computed using the Myers-Miller linear space algorithm [37] which is O(*L*) space and O(*L*^2^) time in the typical sequence length *L*. Given *N *sequences and thus *N*(*N *- 1)/2 = O(*N*^2^) pairs, it is therefore O(*N*^2^*L*^2^) time and O(*N*^2 ^+ *L*) space to construct the distance matrix. The neighbor-joining implementation is O(*N*^2^) space and O(*N*^4^) time, at least up to CLUSTALW 1.82, although O(*N*^3^) time is possible; see e.g. [38]. A single iteration of progressive alignment computes a profile of each subtree from its multiple alignment, which is O(*N*_P_*L*_P_) time and space in the number of sequences in the profile *N*_P _and the profile length *L*_P_, then uses Myers-Miller to align the profiles in O(*L*_P_) space and O(*L*_P_^2^) time. There are *N *- 1 internal nodes in a rooted binary tree and hence O(*N*) iterations. It is often assumed that *L*_P _is O(*L*), i.e. that O(0) gaps are introduced in each iteration. However, we often observe the alignment length to grow approximately linearly, i.e. that O(1) gaps are added per iteration. For example, taking the average over all iterations in all alignments in version 3 of the PREFAB benchmark, Stage 1 of MUSCLE adds 2.8 gaps per iteration to the longer profile. It is therefore more realistic to assume that *L*_P _is O(*L *+ *N*), making one iteration of progressive alignment O(*NL *+ *L*^2^) in both space and time. This analysis is summarized in Table 1.

### Initial distance measure

One might expect (a) that a more accurate distance measure would lead to a more accurate final alignment due to an improved tree, and (b) that errors due to a less accurate distance measure might be eliminated by allowing Stage 2 to iterate more times. Neither of these expectations is supported by our test results (unpublished). Allowing Stage 2 to iterate more than once with the goal of further improving the tree gave no significant improvement with any distance measure. Possibly, the tree is biased towards the MSA that was used to estimate it, and the MSA is biased by the tree used to create it, making it hard to achieve improvements. The most accurate measure on a pair of sequences is presumably the fractional identity *D *computed from a global alignment, but use of *D *in step 1.1 does not improve average accuracy on benchmark tests. The 6-class Dayhoff alphabet used by MAFFT proved to give slightly higher benchmark accuracy scores, despite the fact that other alphabets were found to correlate better with *D *[21]. We also found that the use of the binary approximation *F*^Binary ^gave slightly reduced accuracy scores even when Stage 2 was allowed to iterate. The default choice in MUSCLE is therefore to use the Dayhoff alphabet in step 1.1 and to execute Stage 2 once only. While the impact on the average accuracy of the final alignment due to the different options is not understood, we observe that a better alignment of a pair of sequences is often obtained from a multiple alignment than from a pair-wise alignment, due to the presence of intermediate sequences having higher identities. It is therefore plausible that *D *obtained from the multiple alignment in step 2.1 may be more accurate than *D *obtained from a pair-wise alignment in step 1.1, and this may be relatively insensitive to the method used to create the tree for Stage 1. But this leaves unexplained why *k*-mer counting appears to be as good as or better than *D *in Stage 1. Computing *F *from a pair of sequences is O(*L*) time and O(1) space, so for all pairs the similarity calculation is O(*N*^2^*L*), compared with O(*N*^2^*L*^2^) in CLUSTALW. For a typical *L *around 250, combined with an order of magnitude improvement due to the simplicity of *k*-mer counting compared with dynamic programming, this typically gives a three orders of magnitude speed improvement for computing the distance matrix in MUSCLE compared with CLUSTALW. The default strategy is therefore well justified as a speed optimization, and has the added bonus of providing a small improvement in accuracy.

### Clustering

**Figure 6:**
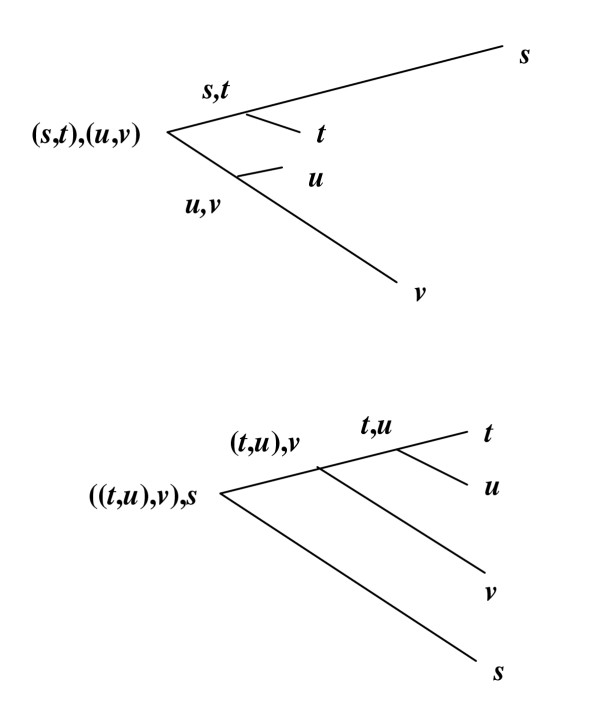
**Neighbor-joining and UPGMA trees for progressive alignment. **Here we show the same set of four sequences and the order in which they will be aligned according to a neighbor-joining tree (above) and a UPGMA tree (below). Notice that *t *and *u *are the most closely related pair, but (*s*, *t*) and (*u*, *v*) are evolutionary neighbors. With neighbor joining, *t *and *u *are not aligned to each other until the root, in contrast to UPGMA, which aligns *s *and *t *as the first pair.

MUSCLE implements both UPGMA and neighbor-joining. We found UPGMA to give slightly better benchmark scores than neighbor-joining; UPGMA is therefore the default option. We expect neighbor-joining to give a better estimate of the correct evolutionary tree (see e.g. [38]). However, it is well-known that alignment accuracy decreases with lower sequence identity (see e.g. [39]). It follows that given a set of profiles, the two that can be aligned most accurately will tend to be the pair with the highest identity, i.e. at the shortest evolutionary distance. This is exactly the pair selected by the nearest-neighbor criterion in UPGMA. By contrast, neighbor-joining selects a pair of evolutionary neighbors, i.e. a pair having a common ancestor. When mutation rates are variable, the evolutionary neighbor may not be the nearest neighbor (Figure 6). This explains why a nearest-neighbor tree may be superior to the true evolutionary tree for guiding a progressive alignment. Neighbor-joining is naively O(*N*^4^) time, although this can be reduced to O(*N*^3^). UPGMA is naively O(*N*^3^) time as the minimum of an *N*^2 ^matrix must be found in each of *N *- 1 iterations. However, this can be reduced to O(*N*^2^) time by maintaining a vector of pointers to the minimum value in each row of the matrix. We are again fortunate to find that the most accurate method is also the fastest.

### Dynamic programming

The textbook algorithm for pair-wise alignment with affine penalties employs three dynamic programming matrices; see e.g. [40,41]. A more time-and space-efficient implementation can be achieved using linear space for the recursion relations and a single matrix for trace-back (Kazutaka Katoh, personal communication). Consider sequences X and Y length *L*_X_, *L*_Y_. We use the following notation: X_*x *_is the *x*th letter in X, X^*x *^the first *x *letters in X, *S*_*xy *_the substitution score (or profile function) for aligning X_*x *_to Y_*y*_, *b*^X^_*x *_the score for a gap-open in Y that is aligned to X_*x*_, *t*^X^_*x *_the score for a gap-close aligned to X_*x*_, *U*_*xy *_the set of all alignments of X^*x *^to Y^*y*^, M_*xy *_the score of the best alignment in *U*_*xy *_ending in a match (i.e., X_*x *_and Y_*y *_are aligned), D_*xy *_the score of the best alignment ending in a delete relative to X (X_*x *_is aligned to an indel) and I_*xy *_the score of the best alignment ending in an insert (Y_*y *_is aligned to an indel). A match is preceded by either a match, delete or insert, so:

M_*xy *_= *S*_*xy *_+ max { M_*x*-1*y*-1_, D_*x*-1*y*-1 _+ *t*^X^_*x*-1_, I_*x*-1*y*-1 _+ *t*^Y^_*y*-1_}     (14)

We assume that a center parameter has been added to *S*_*xy *_such that the gap extension penalty is zero. By considering all possible lengths for the final gap,

D_*xy *_= max(*k*<*x*) [M_*ky *_+ *b*^X^_*k*+1_].     (15)

Here, *k *is the last position in X that is aligned to a letter in Y. Extract the special case of a gap of length 1:

D_*xy *_= max { max(*k*<*x*-1) [M_*ky *_+ *b*^X^_*k*+1_], M_*x*-1*y *_+ *b*^X^_*x*_}.     (16)

Hence,

D_*xy *_= max { D_*x*-1*y*_, M_*x*-1*y *_+ *b*^X^_*x *_}.     (17)

Similarly,

I_*xy *_= max { I_*xy*-1_, M_*xy*-1 _+ *b*^Y^_*y *_}.     (18)

Let the outer loop iterate over increasing *x *and the inner loop over increasing *y*. For fixed *x*, define vectors M^*curr*^_*y *_= M_*xy*_, M^*prev*^_*y *_= M_*x*-1*y*_, D^*curr*^_*x *_= D_*xy*_, D^*prev*^_*x *_= D_*x*-1*y*_; for fixed *x*, *y *define scalars I^*curr *^= I_*xy*_, I^*prev *^= I_*xy*-1_. Now we can re-write (14), (17) and (18) to obtain the following recursion relations:

M^*curr*^_*y *_= S_*xy *_+ max { M^*prev*^_*y*-1_, D^*prev *^_*y*-1 _+ *t*^X^_*x*-1_, I^*prev*^_*y*-1 _+ *t*^Y^_*y*-1 _}     (19)

D^*curr*^_*y *_= max { D^*prev*^_*y*_, M^*prev*^_*y *_+ *b*^X^_*x *_}     (20)

I^*curr *^= max { I^*prev*^, M^*prev*^_*y *_+ *b*^Y^_*y *_}.     (21)

An *L*_X _× *L*_Y _matrix is needed for the trace-back that produces the final alignment.

#### Inner loop

The inner-most dynamic programming loop, which computes the profile function, deserves careful optimization. We will consider the case of PSP; similar optimizations are possible for LE. PSP = Σ_*i *_Σ_*j *_*f *^*x*^_*i *_*f *^*y*^_*j *_*S*_*ij *_= Σ_*i *_*f *^*x*^_*i *_*W*^*y*^_*i*_, where *W*^*y*^_*i *_= Σ_*j *_*f *^*y*^_*j *_*S*_*ij*_. The vector *W*^*y*^_*i *_is used *L*_X _times, and it therefore pays to compute it once and cache it. Observe that a typical profile column contains << 20 different amino acids. We sort the frequencies in decreasing order; the summation Σ_*i *_*f *^*x*^_*i *_*W*^*y*^_*i *_is terminated if a frequency *f *^*x*^_*i *_= 0 is encountered. This typically reduces the time spent in the summation, especially when sequences are closely related. As with *W*^*y*^_*i*_, the sort order is computed once and cached. Observe that the roles of the two profiles are not symmetrical. It is most efficient to choose X, for which frequency sort orders are computed, to be the profile with the lowest amino acid diversity when averaged over columns. With this choice, the summation terminates earlier on average then if the other profile is identified as X. Note that out of *N *- 1 iterations of progressive alignment, a minimum of 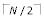 and maximum of *N *- 1 profile-profile alignments will include at least one profile containing one sequence only, and in the refinement phase exactly *N *of the 2*N *- 1 edges in the tree terminate on a leaf. At least half of all profile-profile alignments created in the MUSCLE algorithm therefore include a profile of one sequence only. Special cases where one or both profiles is a single sequence can be handled in separate subroutines, saving overhead due to unneeded loops that are guaranteed to execute once only. This optimization is especially useful for the LE function as it enables the logarithm to be incorporated into the *W *vector.

### Diagonal finding

Many alignment algorithms are optimized for speed, typically at some expense in average accuracy, by using fast methods to identify regions of high similarity between two sequences, which appear as diagonals in the similarity matrix. The alignment path is then constrained to include these diagonals, reducing the area of the dynamic programming matrix that must be computed. MAFFT uses the fast Fourier transform to find diagonals. MUSCLE uses a different technique which we have previously shown [21] have comparable sensitivity and to be significantly faster. We use a compressed alphabet to find *k*-mers in common between two sequences, then attempt to extend the match. In the case of diagonal identification we found compressed alphabets to significantly out-perform the standard amino acid alphabet [21]. Currently, MUSCLE uses 6-mers in the Dayhoff alphabet for diagonal finding, as for the initial distance measure, though other alphabets are known to give slightly better performance [21]. A candidate diagonal is rejected if there is any overlap (i.e., if a single position in one of the sequences appears in two or more diagonals) or if it is less than a minimum length (default 24). The ends of the diagonal are deleted (by default, the first and last five positions) as they are less reliable. Despite these heuristics, we find the use of diagonal-finding to reduce average accuracy and to give only modest improvements in speed for typical input data; this option is therefore disabled by default. Similar results are seen in MAFFT; the most accurate MAFFT script is NWNSI [14], in which diagonal-finding is also disabled.

### Additive profiles

**Figure 7:**
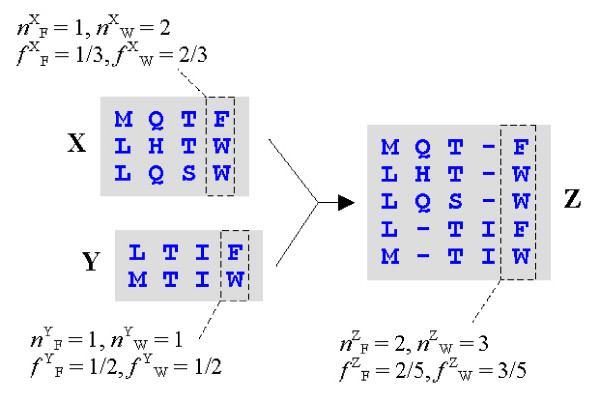
**Additive profiles. **The profile functions in MUSCLE require amino acid frequencies for each column. Here we show the alignment of two profiles X and Y, giving a new profile Z. Note that the count *n*^Z^_*i *_for amino acid *i *in a given column of Z is the sum of the counts in the child profiles, i.e. *n*^Z^_*i *_= *n*^X^_*i *_+ *n*^Y^_*i*_. In terms of frequencies, this becomes *f *^Z^_*i *_= *N*^X^*f *^X^_*i *_/*N*^Z ^+ *N*^Y^*f *^Y^_*i*_/*N*^Z^, where *N*^X^, *N*^Y^, *N*^Z ^are the number of sequences in X, Y and Z respectively. Therefore, given a suitable sequence weighting scheme, it is possible to compute frequencies in Z from the frequencies in X and Y. This avoids the step of building an explicit multiple alignment for Z in order to compute frequencies, as done in CLUSTALW and MAFFT.

Both the PSP and LE profile functions are defined in terms of amino acid frequencies and position-specific gap penalties. The data structure representing a profile is a vector of length *L*_P _in which each element contains frequencies for each amino acid type and a few additional values related to gaps. We call this data structure a *profile vector*, as distinct from a *profile matrix*, an explicit *N *× *L*_P _multiple alignment containing letters and indels. For *N *> 20, using profile vectors reduces the cost of computing the profile function compared with profile matrices, and is therefore preferred for use in dynamic programming. In CLUSTALW and MAFFT, the implementation of progressive alignment builds a profile matrix at each internal node of the tree, which is used to compute a profile vector. This procedure is O(*NL*_P_) = O(*N*^2 ^+ *NL*) in time and space, becoming expensive for large *N*. Observe that the count of a given amino acid in a column in the parent matrix is the sum of the counts in the two child columns that are aligned at that position (Figure 7). With a suitable sequence weighting scheme, it is therefore possible to compute the amino acid frequencies of the parent profile vector from the frequencies in the two child profile vectors and the alignment path. This is an O(*L*_P_) procedure in both time and space, giving a significant advantage for *N *>> 20. Three issues must be addressed to fully implement this idea: the sequence weighting scheme, inclusion of occupancy factors and position-specific gap penalties, and construction of a profile matrix (i.e., the final multiple alignment) at the root node.

#### Sequence weighting

For the frequencies in the parent profile vector to be a linear combination of the child frequencies, the weight assigned to a sequence must be the same in the child and parent profiles. This requirement is not satisfied, for example, by the Henikoff or PSI-BLAST schemes, which compute weights based on a multiple alignment. We therefore choose the CLUSTALW scheme, which computes a fixed weight for each sequence from edge lengths in the tree.

#### Gap representation

To compute gap penalties, we need the frequencies *f*_*o *_of gap opens and *f*_*c *_of gap closes in each position. In the case of the LE profile function, we additionally require the gap frequency *f*_G_. These can be accommodated by storing *f*_*o*_, *f*_*c *_and *f*_*e *_in the profile vector, where *f*_*e *_is the frequency of gap-extensions in the column (meaning that indels are found in a given sequence in the column, the preceding column and in the following column; i.e., a gap-close is not counted as an extension). These three *occupancy frequencies *are sufficient for computing the profile function and the position-specific gap penalties *b *and *t*. Note that we can compute the frequency *f*_G _of indels, as needed for the occupancy factor in the profile function, as follows:

*f*_G _= *f*_*o *_+ *f*_*c *_+ *f*_*e*_.     (22)

**Figure 8:**
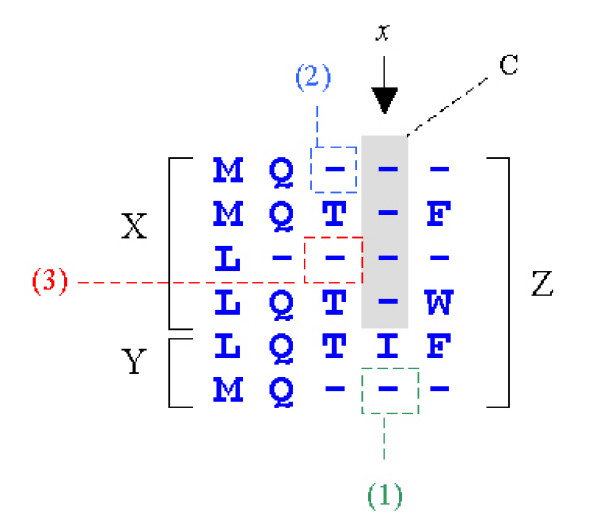
**Occupancy frequencies in additive profiles. **Here we show an alignment of profiles X and Y giving Z. A column C of indels (shaded background) has been inserted at position *x *in order to align X to Y. To compute the number of gap-extensions in column *x *of Z, three cases must be considered: (1) a gap-extension in the corresponding column of Y, (2) a gap-open in the preceding column of X, and (3) a gap-extension in the preceding column of X. By enumerating all such cases, it is straightforward to compute the occupancy frequencies in Z from the occupancy frequencies of X and Y, plus the alignment path.

Now consider the problem of computing the occupancy frequencies in the parent profile vector, given only the child occupancy frequencies and the trace-back path for the alignment. Consider first a diagonal edge in the path, i.e. an edge that does not open or extend a gap, following another diagonal edge. In this case, the occupancy frequencies are computed similarly to amino acid frequencies (as a sum in which a child frequency is weighted according to the total weight of the sequences in its profile). For horizontal or vertical edges, i.e. edges that open or extend gaps, the parent occupancy frequencies can be computed by considering the effect of the new column of indels (Figure 8). It is straightforward to work through all cases and show that the three frequencies *f*_*o*_, *f*_*c *_and *f*_*e *_are sufficient for their values in the parent profile vector to be computed in O(*L*_P_) time from the child profile vectors and alignment path.

#### Construction of the root alignment

By avoiding the use of profile matrices, the complexity of a single progressive alignment iteration is reduced from O(*L*_P _^2 ^+ *NL*_p_) space and O(*L*_P_^2 ^+ *NL*_P_) time to O(*L*_P_^2^) = O(*L*^2 ^+ *NL*) space and time. The *NL *term in the time complexity is now due only to the increase in profile length, and is therefore typically much smaller than before. The root alignment is constructed by storing the alignment path produced at each internal node. For each input sequence, the path to the root is followed, inserting the gaps induced by each alignment path at each internal node. This procedure is O(*NL*_P _log *N*) = O(*N *^2 ^log *N *+ *NL *log *N*) time, and requires O(*NL*_P_) = O(*NL *+ *N *^2^) space for storage of the paths. This is expensive for large *N*, and we therefore optimize this step by using a device we call an *e-string*, a type of edit string.

#### E-strings

**Figure 9:**
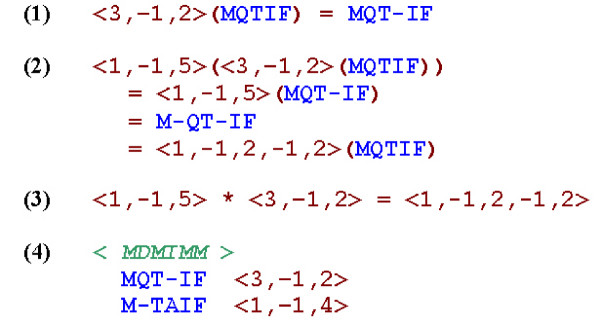
**E-strings. **(1) The effect of the e-string operator <3,-1,2> on the sequence MQTIF. A positive number *n *skips *n *letters, a negative number *-n *insert *n *indels. (2) The effect of applying two successive e-strings. In the last line, the result is expressed as a new e-string applied to the original string. (3) We define multiplication on two e-strings as yielding the e-string that is equivalent to applying the two e-strings in order. (4) An alignment path is conventionally represented as a vector of edge types (*M*, *D *and *I*). In this example, *MDMIMM*, shown above a pairwise alignment, is the path that generates that alignment. The alignment can also be generated by a pair of e-strings (shown to the right). An alignment path is therefore equivalent to a pair of e-strings.

**Figure 10:**
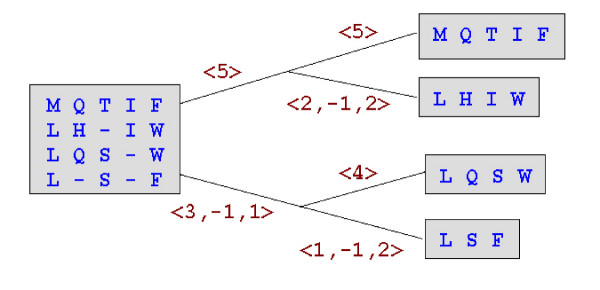
**Root alignment construction. **Here we show the same progressive alignment as Figure 1. Each edge in the tree is labeled with the e-string for its side of the alignment at the parent node. The e-string needed to insert indels into a sequence in the root alignment can be determined by multiplying e-strings along the path to the root. For example, for sequence LSF, the root e-string is <3,-1,1>*<1,-1,2> = <1,-1,1,-1,1>.

An alignment path can be considered as an operator on a pair of sequences that inserts indels into those sequences such that their lengths become equal. Conventionally, an alignment path is represented as a vector of three symbols representing edges in the graph, say M, D and I (for match, delete and insert, i.e. a diagonal, horizontal or vertical edge). Note that indels in one sequence are inserted only by Ds, indels in the other are inserted only by Is. Define an e-string *e *to be a vector of |*e*| integers interpreted as an operator that inserts indels into a string by scanning it from left to right (Figure 9). A positive integer *n *means skip *n *letters of the string; a negative integer -*n *means insert *n *indels at the current position. We require the vector to be in its shortest form, so signs always alternate. We represent an alignment path as a pair of e-strings, one for each sequence, assigned to the appropriate edges in the tree. We will typically find that |*e*|, the length of the e-string, is much less than *L*_P_, the length of the alignment path. Now consider the effect of applying two consecutive e-strings ("multiplying" them). This can be expressed as a third e-string, which can be efficiently computed in O(|*e*|) time from the multiplicands. For each leaf (input sequence), the product is computed of e-strings on the path to the root (Figure 10). The final e-string obtained at the root is then applied to the sequence. This method does not reduce the big-O time or space complexity, but is much faster than a naive implementation.

#### Brenner's method

Steven Brenner (personal communication) observed that a multiple alignment can be alternatively be obtained by aligning each sequence to the root profile. This requires O(*NL*_P_^2^) time, giving a lower asymptotic complexity in *N *at the expense of an additional factor of *L*_P_. This method gives opportunities for errors relative to the "exact" e-string solution (when a sequence misaligns to its copy in the profile), but can also lead to improvements by allowing the sequence to correctly align to conserved motifs that were not apparent when the sequence was added. (Note the resemblance to the refinement stage, which begins by re-aligning individual sequences to the rest). The chances for error are reduced by constraining the alignment to forbid gaps in the root profile. Our tests show that this method gives comparable average accuracy to the e-string solution but to be slower for up to at least a few thousand sequences, and e-strings are therefore used by default.

### Refinement complexity

In the following, we assume that an explicit multiple alignment (profile matrix) of all sequences is maintained, and determine the complexity of each step in Stage 3. Step 3.1 determines the bipartition induced by deleting an edge from the tree. This is O(*N*) time, and sufficiently fast that there is little motivation for further optimization. Step 3.2 extracts profiles for the two partitions from the current multiple alignment and computes their profile vectors, which is O(*NL*_P_) time and space. Step 3.3 performs profile-profile alignment, which is O(*L*_P_^2^) time and space. Step 3.4 computes the SP score, which is O(*N*^2^*L*_P_) time and O(*NL*_P_) space (discussed in more detail shortly). A single iteration of Stage 3 is thus O(*N*^2^*L*_P _+ *L*_P_^2^) time and O(*NL*_P _+ *L*_P_^2^) space. There are O(*N*) edges in the tree, so executing this process for all edges is O(*N*^3^*L*_P _+ *NL*_P_^2^) time and O(*NL*_P _+ *L*_P_^2^) space, which is O(*N*^4 ^+ *N*^3^*L *+ *NL*^2^) time and O(*N*^2 ^+ *NL *+ *L*^2^) space. Assuming that a fixed maximum number of iterations of Stage 3 is imposed, this is also the total complexity of refinement. We now consider optimizations of the refinement stage.

### Anchor columns

A multiple alignment can be divided vertically at high-confidence (*anchor*) columns. Each vertical block is then refined separately, improving speed and reducing space due to the O(*L*^2^) factor in dynamic programming. This strategy has been used by several previous algorithms, including PRRP [13], RASCAL [42] and MAFFT. In MUSCLE, the following criteria are used to identify anchor columns. The profile function (LE or PSP) must exceed a threshold, the averaged profile function over a window around the position must exceed a (lower) threshold, and the column may not contain a gap. In addition, the contribution to the averaged score from a single column has a ceiling, reducing skew in the averaged score due to exceptionally high-scoring columns. These heuristics are designed to avoid anchor columns that have high scores but are either artifacts (similar residues found by chance in unrelated regions) or are too close to variable regions. When performing a profile-profile alignment, each anchor column and its two immediate neighbors (which form the boundaries of vertical blocks) are required to remain aligned; i.e., terminal gaps are forbidden except at the true terminals. Introducing this constraint overcomes a small degradation in average alignment quality that is otherwise observed. This implies that the degradation is sometimes due to cases where a well-conserved region is divided into two parts by an anchor column, one of which becomes short enough that it misaligns to a similar short motif elsewhere.

### SP score

Notice that computation of the SP score dominates the time complexity of refinement and of MUSCLE overall, introducing O(*N*^4^) and O(*N*^3^*L*) terms. We are therefore motivated to consider optimizations of this step. We first consider the contribution SP^a ^to the SP score from amino acids; gap penalties require special treatment. Let *s *and *t *be sequences, *x *be a column, *s *[*x*] be the amino acid of sequence *s *in column *x*, and *S*(*i*, *j*) be the substitution score of amino acids *i *and *j*. It is convenient to impose an (arbitrary) ordering on the sequences and amino acid types. Then,

SP^a ^= Σ_*x *_Σ_*s *_Σ_*t *>*s *_*S*(*s *[*x*], *t *[*x*]).     (23)

Define *δ*(*s*, *i*, *x*) = 1 if *s *[*x*] = *i*, 0 otherwise, and *n*_*i *_[*x*] = Σ_*s *_*δ*(*s*, *i*, *x*). We say *n*_*i *_[*x*] is the *count *of amino acid type *i *in column *x*. We can now transform the sum over pairs of sequences into a sum over pairs of amino acid types:

SP^a ^= Σ_*x *_Σ_*i *_*n*_*i *_Σ_*j *_*n*_*j*>*i *_*S*(*i*, *j*) + 1/2 Σ_*x *_Σ_*i *_(*n*_*i*_^2 ^- *n*_*i*_) *S*(*i*, *i*).     (24)

Frequencies are computed as:

*f *^*x*^_*i *_= *n*_*i *_[*x*]/ *N*.     (25)

Using frequencies,


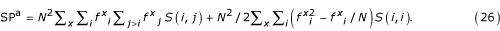


For simplicity, we have neglected sequence weighting; it is straightforward to show that (26) applies unchanged if weighting is used. Note that (23) is O(*N*^2^*L*_P_) but (25) and (26) are O(*NL*_P_). For *N *>> 20, this is a substantial improvement. Let SP^g ^be the contribution of gap penalties to SP, so SP = SP^a ^+ SP^g^. It is natural to seek an O(*NL*_P_) expression for SP^g ^analogous to (26), but to the best of our knowledge no solution is known. Note that in MUSCLE refinement, the absolute value of the SP score is not needed; rather, it suffices to determine the difference in the SP scores before and after re-aligning a pair of profiles. Let SP(*s*, *t*) be the contribution to the SP score from a pair of sequences *s *and *t*, so SP = Σ_*s *_Σ_*t*>*s *_SP(*s*,*t*), and denote the two profiles by X and Y. Then we can decompose SP into intra-and inter-profile terms as follows:

SP = Σ_*s*∈X _Σ_*t*∈X:*t*>*s *_SP(*s*, *t*) + Σ_*s*∈Y _Σ_*t*∈Y:*t*>*s *_SP(*s*, *t*) + Σ_*s*∈X _Σ_*t*∈Y _SP(*s*, *t*)     (27)

Note that the intra-profile terms are unchanged in any alignment that preserves the columns of the profile intact, which is true by definition in profile-profile alignment. This follows by noting that any indels added to align the profiles are guaranteed to be external gaps with respect to any pair of sequences in the same profile. It therefore suffices to compute the change in the inter-profile term:

SP_XY _= Σ_*s*∈X _Σ_*t*∈Y _SP(*s*, *t*).     (28)

This reduces the average time by a factor of about two. We can further improve on this by noting that in the typical case, there are few or no changes to the alignment. This suggests computing the change in SP score by looking only at differences between the two alignments. Let *π*_- _be the alignment path before re-alignment and *π*_+ _the path after re-alignment. The change in alignment can be specified as the set of edges in *π*_- _or *π*_+_, but not both; i.e., by considering a path to be a set of edges and taking the set symmetric difference Δ*π *= (*π*_- _∪ *π*_+_) - (*π*_- _∩ *π*_+_). The path *π*_+ _after re-alignment is available from the dynamic programming traceback. The path *π*_- _before re-alignment can be efficiently computed in O(*L*_P_) time. Note that in order to construct the profile of a subset of sequences extracted from a multiple alignment, those columns that contain only indels in that subset must be deleted. The set of such columns in both profiles is therefore available as a side effect of profile construction, and this set immediately implies *π*_-_. It is a simple O(*L*_P_) procedure to compute Δ*π *from *π*_- _and *π*_+_. Note that SP^a ^is a sum over columns, and there is a one-to-one correspondence between columns and edges in *π*. The change in SP^a ^can therefore be computed as a sum over columns in Δ*π*, with a negative sign for edges from *π*_-_, reducing the time complexity from O(*NL*_P_) to O(*N*|Δ*π*|). We now turn our attention to SP^g^. We say that a gap G *intersects *Δ*π *if and only if any indel in G is in a column in Δ*π*, and denote by Γ the set of gaps that intersect Δ*π*. If a gap does not intersect Δ*π*, i.e. does not have an indel in a changed column, its contribution to SP^g ^is unchanged. It therefore suffices to consider penalties for gaps in Γ, again with negative signs for edges from *π*_-_. The construction of Γ is straightforward in O(*NL*_P_) time. Finally, a sum over pairs in Γ is needed, reducing the O(*N*^2^) component to the smallest possible set of terms.

#### Dimer approximation

**Figure 11:**
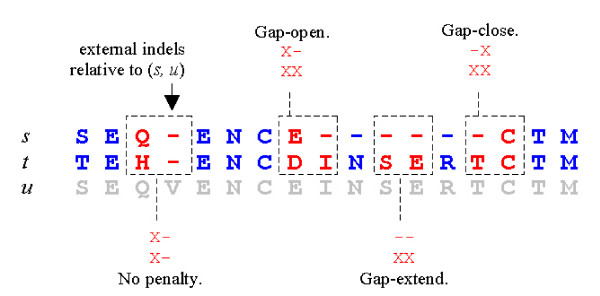
**Dimers in the {X,-} alphabet. **Gap penalties for the sequence pair (*s*, *u*) can be computed be considering all aligned pairs of dimers in the alphabet {X,-}, where X is any amino acid and - is the usual indel symbol. Four cases are highlighted. Note that an aligned pair of identical dimers never contribute a gap penalty as any indels in the dimers are necessarily external, as in the left-most example.

**Figure 12:**
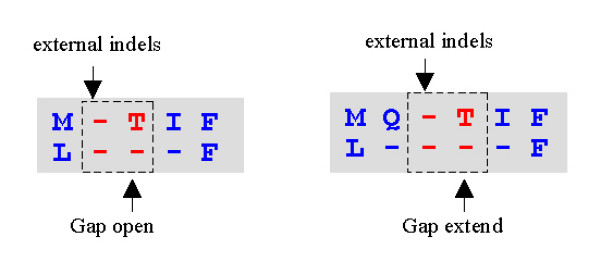
**Problem dimer pair. **The aligned dimer pair -X ↔ -- causes a problem because its gap penalty contribution cannot be computed without additional information. Note that the first column of indels is external; after this column is discarded, different penalties may be needed, as these two examples show.

**Figure 13:**
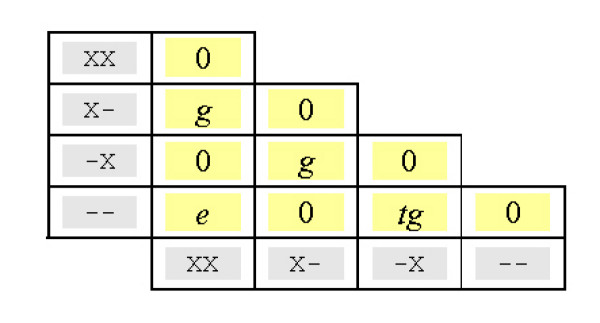
**Dimer substitution matrix. **This matrix specifies the contribution to the total gap penalty for a pair of sequences for each possible pair of aligned dimers. Here, *g *is the per-gap penalty, *e *is the gap-extension penalty. The problem case -X ↔ -- is approximated as *tg*, where *t *is a tunable parameter.

We next describe an approximation to SP that can be computed in O(*NL*_P_) time. Define a two-symbol alphabet {X, -} in which X represents any amino acid and - is the indel symbol. There are four dimers in this alphabet: XX, X-, -X and --, which denote by no-gap, gap-open, gap-close and gap-extend respectively. Re-write a multiple alignment in terms of these dimers, adopting the convention that dimer *ab *composed of symbol *a *in column *x*-1 and symbol *b *in column *x *is written in column *x*. Now consider the contribution to SP^g ^of an aligned pair of dimers, written as *ab*↔*cd*. Clearly XX↔X- adds a gap-open penalty; XX↔-X adds a gap-close (Figure 11). To avoid double-counting, we will include only the penalty contribution of indels in the second column. Then XX↔X- adds a per-gap penalty, but XX↔-X adds zero because the second column does not contain a gap. External indels must be discarded; so, for example, --↔-- adds zero. In fact, aligning two identical dimers always contributes zero because any indel in the second column is found in both sequences and is therefore external. The contribution of all possible pairs of dimers is unambiguous, with the exception of -X↔--, which can add a per-gap or extend penalty (Figure 12). We approximate this case by assigning it a penalty of *tg*, where *g *is the default per-gap penalty and *t *is a tunable parameter, set to 0.2 by default. With this approximation, dimers can be treated like amino acids: the scores for each aligned pair of dimers forms a substitution matrix (Figure 13), and SP^g ^can be computed by summing substitution scores over all pairs of sequences. We can now apply Equation 26, re-interpreting the frequency vectors *f *as having 24 components (20 amino acids and four dimers), and compute the change in SP by considering only those columns in Δ*π*. We find use of the dimer approximation to marginally reduce benchmark scores. By default, MUSCLE therefore uses the exact SP score for *N *≤ 100 and the dimer approximation for *N *> 100, where the higher time complexity of the exact score becomes more noticeable.

### Evaluation of profile functions

**Figure 14:**
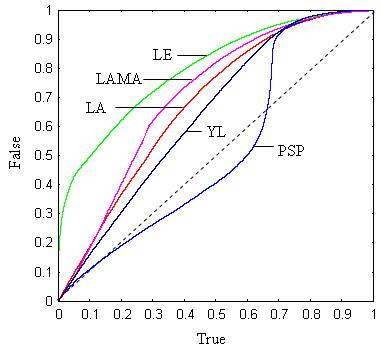
**Discrimination plot for PP2. **The *x *axis is the number of true column pairs with scores ≤ *S *for some value *S*, as a fraction of the total number of true pairs; the *y *axis is the number of false column pairs with scores ≤ *S*, as a fraction of the total number of false pairs. The databases were constructed from the PP2 test set. Shown are discrimination plots for the log-expectation (LE), log-average (LA), Yona-Levitt (YL), LAMA, and profile sum of pairs (PSP) functions. The LE function shows higher discrimination over the entire range of scores than any other function we tested (complete results not shown). The poor performance of the "standard" PSP function is striking. PSP displays negative discrimination over some of its range where it falls below the diagonal (dashed line).

**Figure 15:**
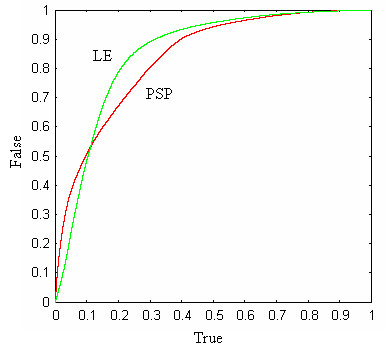
**Discrimination plot for PP. **This is similar to Figure 13, except that the database was generated from the PP test set. Here we see an ambiguous result as the discrimination plots for LE and PSP intersect.

We have previously attempted a systematic comparison of profile functions [30]. The methodology used in that work demanded careful optimization of affine gap parameters for each function. This proved to be time-consuming and tedious, and we therefore tried the following alternative approach, inspired by the notion that a good profile function should be good at discriminating correctly aligned pairs of profile positions from incorrectly aligned pairs. The protocol begins with a set of pair-wise structural alignments. With the sequence of each structure as a query, we used PSI-BLAST to search the NCBI non-redundant protein sequence database [43], giving a multiple sequence alignment (profile) for each structure. Note that we use the term profile in this context to mean the sequence alignment produced by PSI-BLAST, not the scoring matrix. Using the structural alignments as a guide, we then created a database in which columns from the PSI-BLAST profiles were aligned to each other, giving a large set of pairs of alignment columns that we consider to be correctly aligned (the "true" database, although there are undoubtedly misaligned sequences and hence some incorrect pairs). By selecting the same number of pairs of columns at random from structures in different FSSP families, we created a similar ("false") database of unrelated pairs. A profile function was evaluated by computing the score of each pair of columns in both the true and false databases, and then sorting the results in order of increasing score. The results can be displayed by reviewing the sorted list and, for each score *S *in the list, plotting the number of true pairs with score ≤ *S *(*x *axis) against the number of false pairs with score ≤ *S *(*y *axis); we call the resulting graph a *discrimination plot*. Ideally, all true pairs would score higher than all false pairs, in which case the profile function would be a perfect discriminator and would always produce perfect alignments. A function that perfectly discriminates will appear as a Γ-shaped plot; a function that has no ability to discriminate will appear as a diagonal plot along the line *x *= *y*. If a function F has a discrimination plot that is always above another function G (i.e., D_F_(*x*) > D_G_(*x*) ∀ *x*, where D_F _is the discriminator plot for F as a function of *x*), then F has a superior ability to discriminate true from false pairs compared with G. If the plots intersect, the situation is ambiguous and neither function is clearly superior. We used sets of structural alignments from [30] (PP) and [44] (PP2). PP contains 588 structure pairs with sequence identity ≤ 30%, z-score ≥ 15, RMSD ≤ 2.5Å and an alignment length of ≥ 50 positions. These criteria were designed to select pairs of structures with low sequence identity and high structural similarity. PP2 contains 500 pairs selected from the FSSP database [45] with ≤ 30% sequence identity, z-score ≥ 8 and ≤ 12, RMSD ≤ 3.5Å and alignment length ≥ 50. The criteria for PP2 were designed to select challenging alignments with low sequence identity and relatively high structural divergence, leading to a high frequency of gaps and therefore, presumably, a stronger dependence on accurate identification of sequence similarity. Results on PP2 show the LE function to have higher discrimination than all other tested functions (historically, the LE function was designed by systematic trial and error using a wide range of different profile functions with feedback from discrimination plots). This is illustrated in Figure 14, in which the discrimination plot for LE on PP2 is compared with several other functions: PSP, LA, Yona and Levitt's [46], LAMA [47]. Using PP, we again find that LE is superior to LA (not shown), but the comparison with PSP is ambiguous as the discrimination plots intersect (Figure 15). A major advantage of this approach is that no gap penalties are required, with the result that once the databases have been constructed, a new function can be tested in seconds rather than the days or weeks that were needed with the earlier methodology. However, some caveats are in order. We are using PSI-BLAST as a gold standard for creating profiles, but PSI-BLAST may introduce biases both due to its selection of sequences for inclusion in the profile and due to errors in alignments of those sequences to the query. If the profile function will be used to align PSI-BLAST profiles, then this is an appropriate experimental design. But in the case of multiple sequence alignment, where profiles are produced iteratively by the profile function itself, the results may not be directly applicable. We also note that any monotonic transformation of the profile function leaves the discriminator plot unchanged as it does not change the sort order of the scores. (A monotonic transformation is F' = *m*(F) where *m*(*x*) is a monotonically increasing function). However, a monotonic transformation may change the alignments produced by a profile function, so we can regard high discrimination as a necessary but not sufficient condition for a good profile function. One can turn this into a virtue by noting that the discrimination plot allows the relative probability of true versus false to be determined from a score. It is therefore possible to numerically determine a log-odds function from the discrimination plot, which can be evaluated by table look-up. Using discrimination plots for PP2, we found the optimal transformation for LE to be close to linear, in contrast to other functions we tried, including PSP (results not shown). This observation further encouraged us to explore the performance of LE in an MSA algorithm. Testing on multiple alignment benchmarks we find LE to give superior results on BAliBASE, but statistically indistinguishable results on other databases (results not shown). MUSCLE therefore uses LE as the default choice as it sometimes gives better results but has not been observed to give lower average accuracy on any of our tests. It is also useful to introduce a method with a distinctively different scoring scheme as an alternative that may give better results on some input data and may provide unique features for incorporation into jury or consensus systems. One drawback of LE is its relatively slow performance due to the need to compute a logarithm for each cell of the dynamic programming matrix.

### Complexity of MUSCLE

The complexity of MUSCLE is summarized in Table 2. We assume *L*_P _= O(*L *+ *N*), the e-string construction for the root alignment, and a fixed number of refinement iterations.

## Results

MUSCLE offers a variety of options that offer different trade-offs between speed and accuracy. In the following, we report speed and accuracy results for three sets of options: (1) the full MUSCLE algorithm including Stages 1, 2 and 3 with default options; (2) Stages 1 and 2 only, using default options (MUSCLE-prog); and (3) Stage 1 only using the fastest possible options (MUSCLE-fast), which are as follows: *F*^Binary ^is used as a distance measure (Equation 2), the PSP profile function is used, and diagonal finding is enabled.

### Alignment accuracy

In Tables 3 and 4 we report the speed and accuracy of MUSCLE v3.3, CLUSTALW v1.82, Progressive POA, a recently published method that is claimed to be 10 to 30 times faster than CLUSTALW for large alignments [48], and the MAFFT script FFTNS1 v3.82, the fastest previously published method known to us. On the advice of one of the authors of Progressive POA, we used command-line options selecting global alignment with truncated gap scoring (Catherine Grasso, personal communication). We report results both using distance matrices computed by BLAST (POA-blast) and using the distance method built into the program (POA). We use four sets of reference alignments: BAliBASE v2, PREFAB v3, SABmark v1.61, and a version of SMART from July 2000. The accuracy score is *Q*, the number of residue pairs correctly aligned divided by the length of the reference alignment. For more discussion of the reference data, assessment methodology and a comparison of MUSCLE with T-Coffee and NWNSI, the most accurate MAFFT script, see [2].

### Execution speed

**Figure 16:**
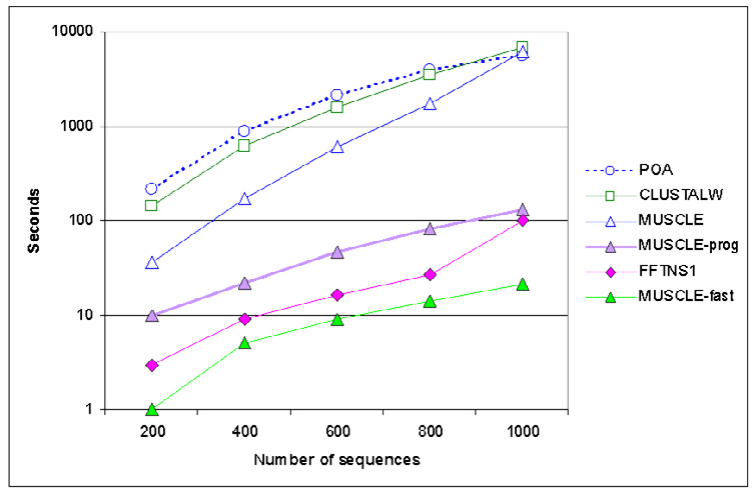
**Execution time as a function of *N*. **This plot shows the execution time as a function of *N *(number of sequences) for the tested alignment methods. Input data is from 200 to 1,000 sequences in increments of 200. Average sequence length is 282, maximum length 454.

To compare speeds for a larger number of sequences, we created a test set by using PSI-BLAST to search the NCBI non-redundant protein sequence database for hits to dienoyl-coa isomerase (1dci in the Protein Data Bank [49]), selecting the highest-scoring 1,000 sequences. This set of sequences had average length 282, maximum length 454 and average pair-wise identity 20%. We aligned randomly chosen subsets of from 200 to 1,000 sequences with each program and noted the total execution time. In the case of 1,000 sequences, the resulting alignments had from 1,100 from 1,400 columns, confirming that it is unrealistic to assume that *L*_P _is O(*L*). Results are shown in Figure 16. We have previously shown that MUSCLE-prog is faster than FFTNS1 on a set of 5,000 sequences, for which we estimated that CLUSTALW would require approximately one year [2]. In this test, MUSCLE-fast is approximately 3× faster than FFTNS1 for 200 sequences, and 5× faster for 1,000 sequences. This trend continues for larger numbers of sequences (complete results not shown), showing that MUSCLE-fast has lower asymptotic complexity, due largely to the use of additive profiles for progressive alignment compared with the profile matrices constructed by FFTNS1.

## Conclusions

MUSCLE demonstrates improvements in accuracy and reductions in computational complexity by exploiting a range of existing and new algorithmic techniques. While the design–typically for practical multiple sequence alignment tools–arguably lacks elegance and theoretical coherence, useful improvements were achieved through a number of factors. Most important of these were selection of heuristics, close attention to details of the implementation, and careful evaluation of the impact of different elements of the algorithm on speed and accuracy. MUSCLE enables high-throughput applications to achieve average accuracy comparable to the most accurate tools previously available, which we expect to be increasingly important in view of the continuing rapid growth in sequence data.

## Availability and requirements

MUSCLE is a command-line program written in a conservative subset of C++. At the time of writing, MUSCLE has been successfully ported to 32-bit Windows, 32-bit Intel architecture Linux, Solaris, Macintosh OSX and the 64-bit HP Alpha Tru64 platform. MUSCLE is donated to the public domain. Source code and executable files are freely available at .

## Figures and Tables

**Table 1:** Complexity of CLUSTALW. Here we show the big-O asymptotic complexity of the elements of CLUSTALW as a function of *L*, the typical sequence length, and *N*, the number of sequences, retaining the highest-order terms in *N *with *L *fixed and vice versa.

**Step**	**O(Space)**	**O(Time)**
Distance matrix	*N*^2 ^+ *L*	*N*^2^*L*^2^
Neighbor joining	*N*^2^	*N*^4^
Progressive (one iteration)	*NL*_P _+ *L*_P _= *NL *+ *L*^2^	*NL*_P _+ *L*_P_^2 ^= *N*^2 ^+ *L*^2^
Progressive (total)	*NL *+ *L*^2^	*N*^3 ^+ *NL*^2^
TOTAL	*N*^2 ^+ *L*^2^	*N*^4 ^+ *L*^2^

**Table 2:** Complexity of MUSCLE. Here we show the big-O asymptotic complexity of the elements of MUSCLE as a function of *L*, the typical sequence length, and *N*, the number of sequences, retaining the highest-order terms in *N *with *L *fixed and vice versa.

**Step**	**O(Space)**	**O(Time)**
*K*-mer distance matrix	*N*^2 ^+ *L*	*N*^2^*L*
UPGMA	*N*^2^	*N*^2^
Progressive (one iteration)	*L*_P_^2 ^= *NL *+ *L*^2^	*L*_P_^2 ^= *N*^2 ^+ *L*^2^
Progressive (root alignment)	*NL*_P _= *N*^2 ^+ *NL*	*NL*_P _log *N *= *N*^2 ^log *N *+ *NL *log *N*
Progressive (*N *iterations + root)	*N*^2 ^+ *NL *+ *L*^2^	*N*^3 ^+ *NL*^2^
Refinement (one edge)	*NL*_P _+ *L*_P_^2 ^= *N*^2 ^+ *L*^2^	*N*^2^*L*_P _+ *L*_P_^2 ^= *N*^3^*+ L*^2^
Refinement (*N *edges)	*N*^2 ^+ *L*^2^	*N*^4^*+ NL*^2^
TOTAL	*N*^2 ^+ *L*^2^	*N*^4 ^+ *NL*^2^

**Table 3:** Accuracy scores. The average accuracy, measured by the *Q *score, is reported for each method on each set of reference alignments.

**Method**	**PREFAB**	**BAliBASE**	**SABmark**	**SMART**
MUSCLE	0.648	0.896	0.430	0.856
MUSCLE-prog	0.634	0.883	0.427	0.837
FFTNS1	0.619	0.844	0.376	0.815
MUSCLE-fast	0.616	0.849	0.396	0.813
CLUSTALW	0.588	0.860	0.404	0.823
POA-blast	0.577	0.839	0.352	0.788
POA	0.576	0.834	0.280	0.797

**Table 4:** CPU times. The total CPU time required to create all alignments in each test set, measured in seconds on a 2.5 GHz Pentium 4 desktop computer.

**Method**	**PREFAB**	**BAliBASE**	**SABmark**	**SMART**
MUSCLE-fast	540	11	45	30
FFTNS1	720	16	70	46
MUSCLE-prog	3,000	52	429	180
MUSCLE	11,000	81	1,500	560
POA-blast	11,000	90	290	670
CLUSTALW	15,000	160	210	480
POA	24,000	130	380	880
